# Supplementary material for: Perception of the threat, mental health burden, and healthcare-seeking behavior change among psoriasis patients during the COVID-19 pandemic
Source: PLoS One. 2021 Dec 9;16(12):e0259852. doi: 10.1371/journal.pone.0259852 (PMC8659332; doi:10.1371/journal.pone.0259852)
Supplement: S1 Table — (DOCX) [file pone.0259852.s002.docx]

**S1 Table**: questionnaire.

**Perception of COVID-19 threats**

**Perceived COVID-19-Related Risk Scale Score for Psoriasis (PCRSP)**

As you may know, people in several countries have recently died from a new disease known as COVID-19. How worried are you or your family about each of the following things -- very worried, somewhat worried, not too worried, not worried, or not worried at all?

**(1) I am worried about acquiring COVID-19.**

1□Not worried at all 2□Not worried 3□Not too worried 4□Somewhat worried 5□Very worried

**(2) I am worried about transmitting COVID-19 to family members if I contract COVID-19.**

1□Not worried at all 2□Not worried 3□Not too worried 4□Somewhat worried 5□Very worried

**(3) I am worried about attending scheduled dermatology appointments for psoriasis due to fear of contracting COVID-19 in hospitals.**

1□Not worried at all 2□Not worried 3□Not too worried 4□Somewhat worried 5□Very worried

**(4) My family is worried about my contact with COVID-19 when visiting dermatology clinics for psoriasis.**

1□Not worried at all 2□Not worried 3□Not too worried 4□Somewhat worried 5□Very worried

**(5) I am worried about using topical agents for psoriasis during the COVID-19 pandemic.**

1□Not worried at all 2□Not worried 3□Not too worried 4□Somewhat worried 5□Very worried

**(6) I am worried about receiving phototherapy for psoriasis due to the possibility of contracting COVID-19 in hospitals/phototherapy centers.**

1□Not worried at all 2□Not worried 3□Not too worried 4□Somewhat worried 5□Very worried 6□I don’t receive phototherapy, thus I have no comments.

**(7) My psoriasis case makes it more likely that I will contract COVID-19**

1□Not worried at all 2□Not worried 3□Not too worried 4□Somewhat worried 5□Very worried

**(8) I am worried about oral drugs or biologics for psoriasis because they probably make me more susceptible to COVID-19 infection.**

1□Not worried at all 2□Not worried 3□Not too worried 4□Somewhat worried 5□Very worried

6□I don’t use oral drugs or biologics for psoriasis, so I have no comments.

**(9) My family is worried about oral drugs or biologics for psoriasis because they probably make me more susceptible to COVID-19 infection.**

1□Not worried at all 2□Not worried 3□Not too worried 4□Somewhat worried 5□Very worried

6□I don’t oral drugs or biologics for psoriasis, thus no comments.

**(10) I am worried about COVID-19 making my psoriasis worse if I am unlucky enough to catch the infection.**

1□Not worried at all 2□Not worried 3□Not too worried 4□Somewhat worried 5□Very worried

**(11) I am worried about having a higher chance of getting a serious illness or dying due to underlying psoriasis if infected with COVID-19.**

1□Not worried at all 2□Not worried 3□Not too worried 4□Somewhat worried 5□Very worried

**(12) I am worried about the COVID-19 pandemic destroying our healthcare system and decreasing my accessibility to medical consultation and care for psoriasis.**

1□Not worried at all 2□Not worried 3□Not too worried 4□Somewhat worried 5□Very worried

**(13) I am worried about drug shortages for psoriasis therapy amidst the COVID-19 pandemic.**

1□Not worried at all 2□Not worried 3□Not too worried 4□Somewhat worried 5□Very worried

**(14) Please choose the top three items that you are most concerned with from the questions mentioned above.**

( ________, ________ , ________ )

**Total score = ____________________(Add the scores for questions 1-13)**

**Psychological Aspects**

**Depressive, anxiety, insomnia, and stress-related symptoms (DAISS)**

1. **I have felt depressed more frequently during the COVID-19 pandemic than before the outbreak.**

1□Strongly Disagree 2□Disagree 3□Neither Agree nor Disagree 4□Agree 5□Strongly Agree

(2) **I have had more panic, trembling of hands, fear, breathing difficulty, a sense of increased heart rate, or heart missing a beat during the COVID-19 pandemic than before the outbreak.**

1□Strongly Disagree 2□Disagree 3□Neither Agree nor Disagree 4□Agree 5□Strongly Agree

1. **I have suffered from insomnia symptoms more frequently during the COVID-19 pandemic than before the outbreak.**

1□Strongly Disagree 2□Disagree 3□Neither Agree nor Disagree 4□Agree 5□Strongly Agree

1. **I have felt exhausted, agitated, had difficulty winding down, and had difficulty relaxing more frequently during the COVID-19 pandemic than before the outbreak.**

1□Strongly Disagree 2□Disagree 3□Neither Agree nor Disagree 4□Agree 5□Strongly Agree

**Impact of Event Scale-Revised**

Below is a list of difficulties people sometimes have after stressful life events. Please read each item and then indicate how much you have been distressed or bothered by these difficulties concerning COVID-19.

Item Response Anchors are 0 = Not at all, 1 = A little bit, 2 = Moderately, 3 = Quite a bit, 4 = Extremely

1. **Any reminder has brought back feelings about COVID-19.**

0□Not at all 1□A little bit 2□Moderately 3□Quite a bit 4□Extremely.

1. **I have had trouble staying asleep.**

0□Not at all 1□A little bit 2□Moderately 3□Quite a bit 4□Extremely.

1. **Other things have kept me thinking about COVID-19.**

0□Not at all 1□A little bit 2□Moderately 3□Quite a bit 4□Extremely.

1. **I have felt irritable and angry.**

0□Not at all 1□A little bit 2□Moderately 3□Quite a bit 4□Extremely.

1. **I have avoided letting myself get upset when I think about COVID-19, or when I am reminded of COVID-19.**

0□Not at all 1□A little bit 2□Moderately 3□Quite a bit 4□Extremely.

1. **I have thought about COVID-19 when I didn’t mean to.**

0□Not at all 1□A little bit 2□Moderately 3□Quite a bit 4□Extremely.

1. **I have felt as if the COVID-19 pandemic hadn’t happened or wasn’t real.**

0□Not at all 1□A little bit 2□Moderately 3□Quite a bit 4□Extremely.

1. **I have stayed away from things that remind me about COVID-19.**

0□Not at all 1□A little bit 2□Moderately 3□Quite a bit 4□Extremely.

1. **Pictures about COVID-19 have popped into my mind.**

0□Not at all 1□A little bit 2□Moderately 3□Quite a bit 4□Extremely.

1. **I have been jumpy and easily startled.**

0□Not at all 1□A little bit 2□Moderately 3□Quite a bit 4□Extremely.

1. **I have tried not to think about COVID-19.**

0□Not at all 1□A little bit 2□Moderately 3□Quite a bit 4□Extremely.

**(12) I have been aware that I still have a lot of feelings about COVID-19, but I have not dealt with them.**

0□Not at all 1□A little bit 2□Moderately 3□Quite a bit 4□Extremely.

1. **My feelings about COVID-19 have been kind of numb.**

0□Not at all 1□A little bit 2□Moderately 3□Quite a bit 4□Extremely.

1. **I have found myself acting or feeling like I was back at the time of COVID-19.**

0□Not at all 1□A little bit 2□Moderately 3□Quite a bit 4□Extremely.

1. **I have had trouble falling asleep.**

0□Not at all 1□A little bit 2□Moderately 3□Quite a bit 4□Extremely.

1. **I have had waves of strong feelings about COVID-19.**

0□Not at all 1□A little bit 2□Moderately 3□Quite a bit 4□Extremely.

1. **I have tried to remove COVID-19 from my memory.**

0□Not at all 1□A little bit 2□Moderately 3□Quite a bit 4□Extremely.

1. **I have had trouble concentrating.**

0□Not at all 1□A little bit 2□Moderately 3□Quite a bit 4□Extremely.

1. **Reminders of COVID-19 have caused me to have physical reactions, such as sweating, trouble breathing, nausea, or a pounding heart.**

0□Not at all 1□A little bit 2□Moderately 3□Quite a bit 4□Extremely.

1. **I have had dreams about COVID-19.**

0□Not at all 1□A little bit 2□Moderately 3□Quite a bit 4□Extremely.

1. **I have felt watchful and on guard.**

0□Not at all 1□A little bit 2□Moderately 3□Quite a bit 4□Extremely.

**(22) I have tried not to talk about COVID-19.**

0□Not at all 1□A little bit 2□Moderately 3□Quite a bit 4□Extremely.

**Behavior Changes**

Please read each item and then indicate whether you practiced the following behaviors more frequently during the COVID-19 pandemic than before the outbreak.

**(1) Postponed/interrupted/cancelled/decreased clinic visits for psoriasis.**

1□Strongly Disagree 2□Disagree 3□Neither Agree nor Disagree 4□Agree 5□Strongly Agree

1. **Postponed/interrupted/cancelled/decreased phototherapy for psoriasis.**

1□Strongly Disagree 2□Disagree 3□Neither Agree nor Disagree 4□Agree 5□Strongly Agree 6□I Don’t Receive Phototherapy

1. **Asked for a shift towards a prolonged prescription for my psoriasis or switching to drugs with longer action to reduce return clinic visits.**

1□Strongly Disagree 2□Disagree 3□Neither Agree nor Disagree 4□Agree 5□Strongly Agree

1. **Postponed/interrupted/discontinued/decreased oral drugs for psoriasis.**

1□Strongly Disagree 2□Disagree 3□Neither Agree nor Disagree 4□Agree 5□Strongly Agree 6□I Don’t Receive Oral Drugs For My Psoriasis.

1. **Postponed/interrupted/discontinued/decreased biologics for psoriasis.**

1□Strongly Disagree 2□Disagree 3□Neither Agree nor Disagree 4□Agree 5□Strongly Agree 6□I Don’t Receive Biologics For My Psoriasis.

1. **Not taken my medications for psoriasis, according to doctors’ prescriptions or instructions because of the COVID-19 threat.**

1□Strongly Disagree 2□Disagree 3□Neither Agree nor Disagree 4□Agree 5□Strongly Agree

1. **The fears of COVID-19 deterred me from seeking healthcare or providers offering medical services or consultations for other non-COVID-19 diseases.**

1□Strongly Disagree 2□Disagree 3□Neither Agree nor Disagree 4□Agree 5□Strongly Agree
