# Supplementary material for: Perception of the threat, mental health burden, and healthcare-seeking behavior change among psoriasis patients during the COVID-19 pandemic
Source: PLoS One. 2021 Dec 9;16(12):e0259852. doi: 10.1371/journal.pone.0259852 (PMC8659332; doi:10.1371/journal.pone.0259852)
Supplement: S2 Table — (DOCX) [file pone.0259852.s003.docx]

**S2 Table.** Perception of the COVID-19 threat among patients with psoriasis.

| **Item** | **Score (Mean ± SD)** |
| --- | --- |
| Perception of the COVID-19 threat |  |
| (1) worry about acquiring COVID-19 | 3.36 ± 1.20 |
| (2) worry about transmitting COVID-19 to family members | 3.95 ± 1.12 |
| (3) worry about attending scheduled dermatology appointments for psoriasis due to fear of contracting COVID-19 in hospitals | 3.32 ± 1.09 |
| (4) family worries about my contact with COVID-19 when visiting dermatology clinics for psoriasis, median (IQR) | 3.26 ± 1.09 |
| (5) worry about using topical agents for psoriasis during the COVID-19 pandemic | 2.04 ± 0.94 |
| (6) worry about receiving phototherapy for psoriasis due to the possibility of contracting COVID-19 in hospitals/phototherapy centers | 2.50 ± 1.14 |
| (7) psoriasis makes it more likely that I will contract COVID-19 | 2.62 ± 1.16 |
| (8) worry about oral drugs or biologics for psoriasis because they probably make me more susceptible to COVID-19 infection | 2.52 ± 1.12 |
| (9) family worries about oral drugs or biologics for psoriasis because they probably make me more susceptible to COVID-19 infection | 2.39 ± 1.05 |
| (10) worry about COVID-19 making my psoriasis worse if I am unlucky enough to catch the infection | 2.94 ± 1.20 |
| (11) worry about having a higher chance of getting a serious illness or dying due to underlying psoriasis if infected with COVID-19 | 3.03 ± 1.21 |
| (12) worry about the COVID-19 pandemic destroying our healthcare system and decreasing my accessibility to medical consultation and care for psoriasis | 3.10 ± 1,22 |
| (13) worry about drug shortages for psoriasis therapy amidst the COVID-19 pandemic | 3.12 ± 1.24 |
| average PCRSP scores | 2.97 ± 0.80 |

SD, standard deviation; PCRSP, Perceived COVID-19-Related Risk Scale score for Psoriasis
