# Supplementary material for: Perception of the threat, mental health burden, and healthcare-seeking behavior change among psoriasis patients during the COVID-19 pandemic
Source: PLoS One. 2021 Dec 9;16(12):e0259852. doi: 10.1371/journal.pone.0259852 (PMC8659332; doi:10.1371/journal.pone.0259852)
Supplement: S3 Table — (DOCX) [file pone.0259852.s004.docx]

**S3 Table**. Psychological impact and behavioral changes caused by the COVID-19 pandemic on patients with psoriasis.

| **Item** | **Score (Mean ± SD)** |
| --- | --- |
| Depressive, anxiety, insomnia, and stress-related symptoms (DAISS) |  |
| 1. felt depressed more frequently during the COVID-19 pandemic than before the outbreak | 2.31 ± 1.09 |
| 1. had more panic, trembling of hands, fear, breathing difficulty, a sense of increased heart rate, or heart missing a beat during the COVID-19 pandemic than before the outbreak. | 1.95 ± 0.93 |
| (3) have suffered from insomnia symptoms more frequently during the COVID-19 pandemic than before the outbreak. | 1.92 ± 0.94 |
| (4) felt exhausted, agitated, had difficulty winding down, and difficulty relaxing more frequently during the COVID-19 pandemic than before the outbreak. | 2.05 ± 1.0 |
| Impact of Event Scale-Revised | 14.40 ± 12.38 |
| Healthcare-seeking behavior changes |  |
| 1. Postponed/interrupted/cancelled/decreased clinic visits for psoriasis | 2.62 ± 0.92 |
| 1. Postponed/interrupted/cancelled/decreased phototherapy for psoriasis | 2.22 ± 0.95 |
| 1. Asked for a shift towards a prolonged prescription for my psoriasis or switching to drugs with longer action to reduce return clinic visits | 3.16 ± 0.93 |
| (4) Postponed/interrupted/discontinued/decreased oral drugs for psoriasis | 2.33 ± 0.81 |
| (5) Postponed/interrupted/discontinued/decreased biologics for psoriasis | 2.41 ± 0.86 |
| (6) Not taken my medications for psoriasis, according to doctors’ prescriptions or instructions because of COVID-19 threat | 2.32 ± 0.89 |
| (7) The fears of COVID-19 deterred me from seeking healthcare or providers offering medical services or consultations for other non-COVID-19 diseases | 2.36 ± 0.85 |
