# Supplementary material for: Perception of the threat, mental health burden, and healthcare-seeking behavior change among psoriasis patients during the COVID-19 pandemic
Source: PLoS One. 2021 Dec 9;16(12):e0259852. doi: 10.1371/journal.pone.0259852 (PMC8659332; doi:10.1371/journal.pone.0259852)
Supplement: Attachment — Submitted filename: REBUTTAL LETTER.docx [file pone.0259852.s006.docx]

**REBUTTAL LETTER**

**Dear Editor,**

On behalf of all the co-authors, we thank you for allowing us to revise our manuscript entitled **“****Perception of the threat, mental health burden, and healthcare-seeking behavior change among psoriasis patients during the COVID-19 pandemic (PONE-D-21-15316R1).”** The manuscript has been revised according to the suggestions and comments from the editors and reviewers. The changes in the manuscript are highlighted in red. We also submitted both the final (clean) version and the marked-up (track changes/highlighted) version. Our point-by-point responses to reviewers’ comments are listed below. We have also revised the title page following the instructions. We thank you for the constructive criticism; we hope this revised version will be acceptable for publication in PLOS ONE.

Yours sincerely,

**Yu-Huei Huang**

Department of Dermatology, Chang Gung Memorial Hospital, Linkou Branch, Taoyuan, Taiwan

No.5, Fuxing St., Guishan Dist., Taoyuan City 333, Taiwan (R.O.C.)

Tel: 886- 2-27135211ext 3397; Fax: 886- Fax: 02-27191623

E-mail: huangyh@adm.cgmh.org.tw

**Journal Requirements**

Please review your reference list to ensure that it is complete and correct. If you have cited papers that have been retracted, please include the rationale for doing so in the manuscript text or remove these references and replace them with relevant current references. Any changes to the reference list should be mentioned in the rebuttal letter that accompanies your revised manuscript. If you need to cite a retracted article, indicate the article’s retracted status in the References list and also include a citation and full reference for the retraction notice.

**Response 1**:

Thank you for the instructions. We have reviewed our reference list, and no cited papers have been retracted.

**Reviewers’ comments**

**Reviewer #1:**

Comment#1: Although much better, English editing in several places is still necessary. Just one example in lines 294-5 is provided here: Psoriasis patients with are more prone to develop comorbidities.....

**Response 1**: Thank you for the comments. We have rephrased this sentence as follows: Patients suffering from psoriasis are more likely to face the risks of comorbidities, such as hypertension, diabetes, and cardiovascular disease. The manuscript has been carefully reviewed by an experienced editor whose first language is English and who specializes in editing papers written by scientists whose native language is not English. The English editing certificate is provided below.
